# Words and Things

**Published:** 1918-02-23

**Authors:** T. C. Mackenzie

**Affiliations:** Medical Superintendent. District Asylums, Inverness.


*To the Editor of* THE HOSPITAL.

### "Words and Things."

*To the Editor of* THE HOSPITAL.

SIR,—Your issue of February 9 contains an article under the above heading, the greater part of which I have read with interest, and with warm approval in the case of the sentences that contain the admonition that the public should be educated to regard mental diseases as comparable, in large part at least, with other diseases. The concluding sentences, however, are responsible for very different feelings. The writer of your article has either utterly failed to understand the expressed views of Scottish alienists, or has deliberately misrepresented them. Alienists in Scotland have not failed to take advantage of the medium provided in their annual reports for the education of the public on the problems of madness, or lunacy, or insanity, or mental disease. A study of the annual reports of the Scottish asylums would provide any serious reader with a demonstration of this fact, and I strongly recommend the writer of your article to make such an investigation and study before he again expresses his views in such sweeping terms on the attitude of alienists to the problems of mental diseases. Should he not be in a position readily to obtain such annual reports, I shall be glad to furnish him with a few.—I am, yours truly,

District Asylums, Inverness.
February 13, 1918.

T. C. MACKENZIE,
Medical Superintendent.

⁂ We shall be pleased to receive the reports alluded to by Dr. Mackenzie, and shall not fail to make amends to the Scottish alienists if we find that the character of the reports requires it. But why do they hide their light under whatever Scotch measure answers to the English bushel? We are always pleased to receive the annual reports of asylums, and to comment on anything of public interest they contain. The difficulty often has been to find anything of public interest in them. Our readers would scarcely be interested in masses of statistics setting forth the average number daily resident, the number and kinds of garments made and mended, and so forth. Our feeble arms cannot toss such an unwieldy caber.—ED. THE HOSPITAL.

